# Immunochemical Recognition of *Bothrops rhombeatus* Venom by Two Polyvalent Antivenoms

**DOI:** 10.3390/toxins16030152

**Published:** 2024-03-15

**Authors:** Karen Sarmiento, Jorge Zambrano, Carlos Galvis, Álvaro Molina-Olivares, Marisol Margarita Villadiego-Molinares, Johanna Alejandra Ramírez-Martínez, Ana Lucía Castiblanco, Fabio A. Aristizabal

**Affiliations:** 1 Department of Physiological Sciences, Faculty of Medicine, Pontificia Universidad Javeriana, Bogotá 110231, Colombia; 2 School of Agricultural, Livestock, and Environmental Sciences ECAPMA, Universidad Nacional Abierta y a Distancia, Bogotá 111511, Colombia; jazambranon@gmail.com; 3 Department of Fauna Collection Cali Zoo, Fundación Zoológica de Cali, Cali 760045, Colombia; carlos.galvis@fzc.com.co; 4 Faculty of Medicine, Pontificia Universidad Javeriana, Bogotá 110231, Colombia; aa_molinao@javeriana.edu.co; 5 Fundación para la Gestión del Riesgo, Bogotá 111071, Colombia; mvilladiego@gestiondelriesgo.org (M.M.V.-M.); jramirez@gestiondelriesgo.org (J.A.R.-M.); 6 Instrumental Analysis Laboratory, Biotechnology Institute, Universidad Nacional de Colombia, Bogotá 111321, Colombia; alcastiblancor@unal.edu.co; 7 Department of Pharmacy, Faculty of Science, Biotechnology Institute, Universidad Nacional de Colombia, Bogotá 111321, Colombia; faaristizabalg@unal.edu.co

**Keywords:** antiophidic antivenom, antivipmyn-Tri, *Bothrops asper*, *Bothrops rhombeatus*, National Institute of Health, snake bite

## Abstract

The protein profile of *Bothrops rhombeatus* venom was compared to *Bothrops asper* and *Bothrops atrox,* and the effectiveness of antivenoms from the National Institute of Health of Colombia (INS) and Antivipmyn-Tri (AVP-T) of Mexico were analyzed. Protein profiles were studied with sodium dodecyl sulfate–polyacrylamide gel electrophoresis (SDS-PAGE) and reverse-phase high-performance liquid chromatography (RP-HPLC). The neutralizing potency and the level of immunochemical recognition of the antivenoms to the venoms were determined using Western blot, affinity chromatography, and enzyme-linked immunosorbent assay (ELISA). Bands of phospholipase A2 (PLA2), metalloproteinases (svMPs) I, II, and III as well as serine proteinases (SPs) in the venom of *B. rhombeatus* were recognized by SDS-PAGE. With Western blot, both antivenoms showed immunochemical recognition towards PLA2 and svMP. INS showed 94% binding to *B. rhombeatus* venom and 92% to *B. asper* while AVP-T showed 90.4% binding to *B. rhombeatus* venom and 96.6% to *B. asper*. Both antivenoms showed binding to PLA2 and svMP, with greater specificity of AVP-T towards *B. rhombeatus.* Antivenom neutralizing capacity was calculated by species and mL of antivenom, finding the following for INS: *B. asper* 6.6 mgV/mL, *B. atrox* 5.5 mgV/mL, and *B. rhombeatus* 1.3 mgV/mL. Meanwhile, for AVP-T, the following neutralizing capacities were found: *B. asper* 2.7 mgV/mL, *B. atrox* 2.1 mgV/mL, and *B. rhombeatus* 1.4 mgV/mL. These results show that both antivenoms presented similarity between calculated neutralizing capacities in our trial, reported in a product summary for the public for the *B. asper* species; however, this does not apply to the other species tested in this trial.

## 1. Introduction

Snake bites, or ophidism, are a global public health problem, considered a neglected tropical disease (NTD) in WHO category A [1]. Worldwide, it is estimated that 5.4 million people are bitten by snakes annually, producing envenomation or ophidiotoxicosis in between 1.8 and 2.7 million, mortality in between 81,410 and 137,880, and about three times as many amputations and disabilities [1]. 

In Colombia, there are two families of venomous snakes of medical importance: *Viperidae*, containing the genera *Crotalus, Bothriopsis, Bothrocophias, Bothrops Lachesis*, and *Porthidium*; the family *Elapidae* includes, for Colombia, approximately 29 species of snakes of the genus *Micrurus* known as true corals [2]. According to the National Institute of Health, in 2021, 4702 clinically confirmed cases of ophidism were reported, calculating a cumulative national incidence rate of 9.2/100,000 inhabitants and a cumulative lethality of 0.57 [3]. The INS epidemiological bulletins for the year 2022 showed, in the last three periods, an increase in the number of cases between 6 and 19% compared to the same periods of the previous year. At the national level, it is estimated that 65–74% of cases are caused by snakes of the *Viperidae* family, 1–3% by snakes of the *Elapidae* family, and 20–25% of the species of snake involved are not identified.

**Figure 1:**
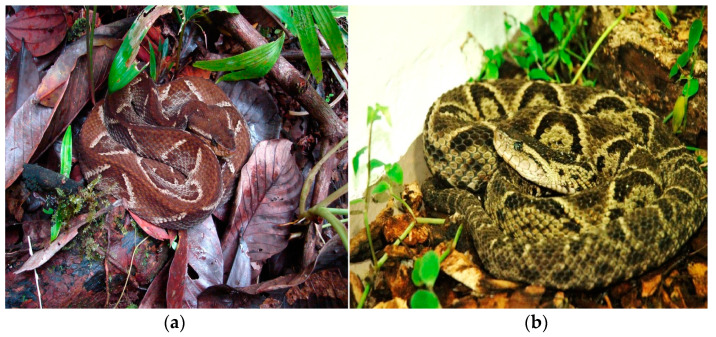
Photographs of the species *B. asper* (**a**) and *B. rhombeatus* (**b**). Courtesy: Carlos Galvis 2019, Fundación Zoológica de Cali.

Between 50 and 80% of snake bite accidents in Colombia are recorded under the name *Bothrops asper* [4] (Figure 1a) due to its distribution in the country and its high reactivity to disturbance by humans; however, there are two or more species with similar morphological characteristics with which it can be confused. One of these is *Bothrops rhombeatus* (Figure 1b) [5], located in the Andean region of Colombia, and given that until recently the composition of its venom was unknown, it is not possible to predict the similarity or variation in the presentation of the clinical picture concerning the other species of the genus *Bothrops*. Additionally, *Bothrops atrox* is one of the species from the *Bothrops* genus more common in the country, so it is easily confused with *B. asper*. The intraspecific variability in snake venom resulting in the variability in the patient’s syndromic presentation deserves special consideration because the treatment and clinical management are highly specific and personalized [6].

The antiophidic antivenom is the only drug that has proven to be effective in neutralizing the toxic effect of the venom [7,8]. Antiophidic antivenoms composed of purified immunoglobulins are produced and marketed in Colombia. However, due to the limited production of this antivenom by the National Institute of Health, there is a permanent shortage of the product. For this reason, the Colombian Ministry of Health and Social Protection declared a state of sanitary emergency in 2010 [9,10], which has allowed the classification of antivenom as an essential medicine with the consequent importation of antivenoms from producing countries. The antivenoms marketed in Colombia come from the National Health Institute (INS). Of the imported antivenoms, those produced by SILANES, a Mexican company that produces the antivenom Antivipmyn-Tri (AVP-T), are noteworthy. The two antivenoms are polyvalent for the *Viperidae* family and differ in the immunoglobulin production process and in their neutralizing capacity. The INS antivenom is a mixture of complete immunoglobulins. One vial of 10 mL has a neutralizing capacity of 10 mg of *Crotalus* sp. venom and 70 mg of *Bothrops* sp. venom [11]. On the other hand, one vial of 10 mL of AVP-T is composed of purified F(ab’)2 fragments with a neutralizing capacity of 780 LD50 (30 mg) of dehydrated *Crotalus* sp. venom and 200 LD50 (15 mg) of dehydrated *Lachesis* sp. venom [12,13]. 

Therefore, taking into account the importance of the recognition of new species of *Bothrops* sp. in our environment and the use of antiophidic antivenoms, our objective was to assess the immunochemical recognition of the venom of *B. rhombeatus, B. asper, and B. atrox* by INS and AVP-T antivenoms, associated with their neutralizing capacity.

## 2. Results

### 2.1. Protein Profile of Venoms and Antivenoms

**Figure 2:**
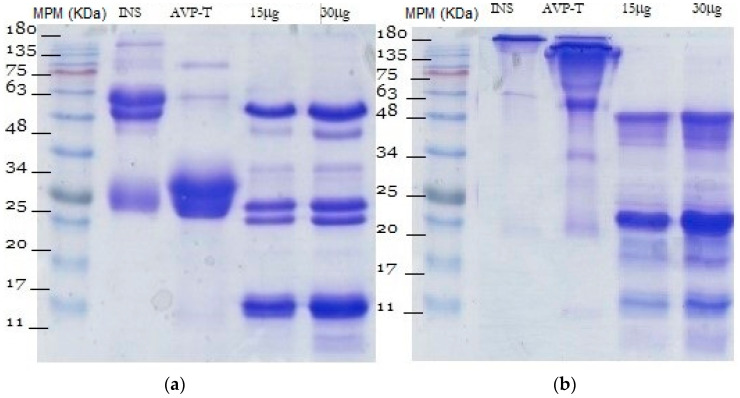
Electrophoresis of *B. rhombeatus* venom and antivenoms. Reducing conditions (**a**) and non-reducing conditions (**b**). MPM: molecular weight markers; INS: antivenom from the National Institute of Health; AVP-T: Antivipmyn-Tri antivenom; 15 μg: 15 μg of *B. rhombeatus* venom; 30 μg: 30 μg of *B. rhombeatus* venom.

The electrophoretic profiles of INS and AVP-T antivenoms and *B. rhombeatus* venom are shown in Figure 2. The electrophoretic and chromatographic analytical methods are used to make a qualitative comparison of the venoms’ protein compositions. Some authors state the importance of electrophoretic and chromatographic analytical results as a step before neutralizing capacity analysis or coupling essays, even before antivenom production, in order to perform an evaluation of their purity, optimize the antivenoms’ production, and identify new bioactive components [14]. To corroborate the protein fractions found, the results are compared with global databases where the molecular weight of snake venom proteins has been standardized, e.g., www.rcsb.org (accesed on 15 January 2022) www.uniprot.org (accesed on 20 July 2021).

Under reducing conditions, INS antivenom showed conspicuous protein bands between 48 and 70 kDa and bands of lower intensity between 25 and 30 kDa; in addition, a band at 70 kDa and another around 170 kDa indicated the presence of high-molecular-weight aggregates [14], while, under non-reducing conditions, INS antivenom showed a band of 180 kDa. The AVP-T antivenom showed a large band between 20 and 30 kDa and two other bands of lower intensity at 63 and 75 kDa under reducing conditions (Figure 2).

**Figure 3:**
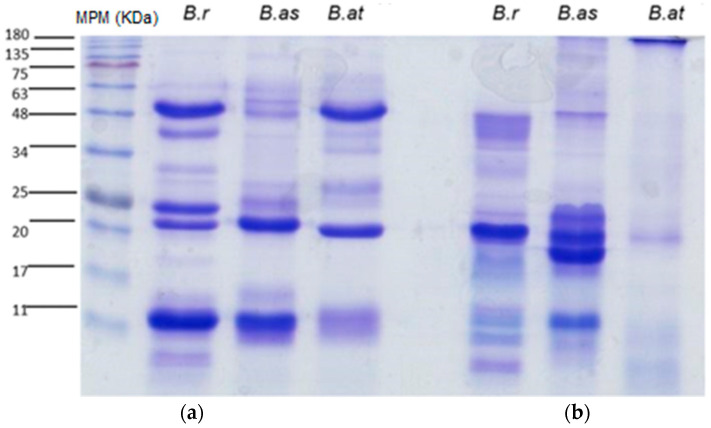
Electrophoretic pattern of *B. rhombeatus, B. asper,* and *B. atrox.* Reducing conditions (**a**) and non-reducing conditions (**b**). MPM: molecular weight markers; B.r: 15 μg of *Bothrops rhombeatus* venom; B.as: 15 μg of *Bothrops asper* venom; B.at: 15 μg of *Bothrops atrox* venom.

In Figure 2 and Figure 3, the venom of *B. rhombeatus* showed four notable bands of 48, 25, 20, and 11 kDa and five bands of lower intensity of 63, 45, 34, 30, and 9 kDa. In Figure 3, the electrophoretic profiles of the venom of *B. rhombeatus*, *B. asper*, and *B. atrox* were compared under both reducing and non-reducing conditions. Under reducing conditions, *B. asper* venom showed two high-intensity bands of 20 and 11 kDa, a series of lower-intensity bands between 48 and 75 kDa, and three other bands of 30, 20, and 15 kDa of low intensity. The *B. atrox* venom showed two high-intensity bands at 48 and 20 kDa and lower-intensity bands at 75, 40, 34, 30, 30, 28, and 11 kDa. However, under non-reducing conditions, the venoms showed different profiles.

**Figure 4:**
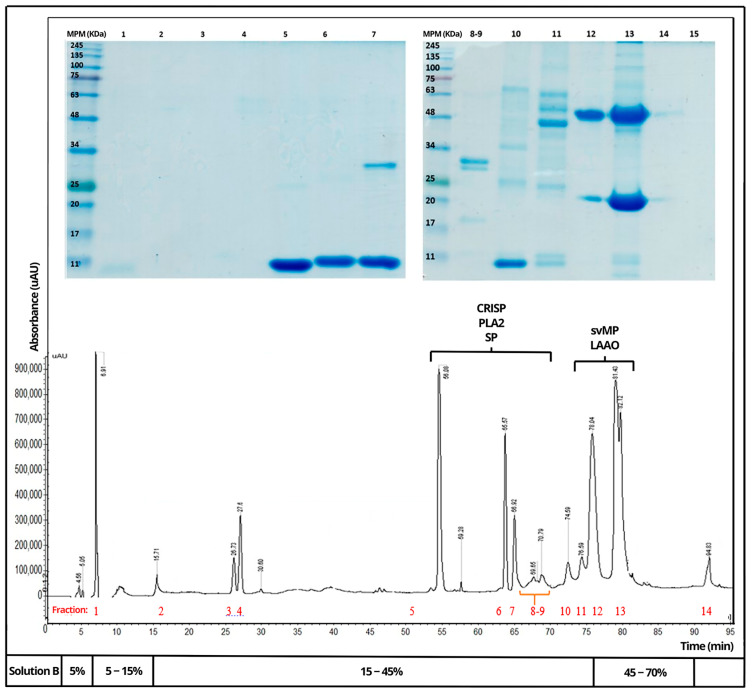
Reverse-phase HPLC separation of the venom proteins from *Bothrops rhombeatus*. Insert: SDS–PAGE of the isolated chromatographic fractions run under non-reducing conditions (Red numbers represent the fractions selected for SDS—PAGE and affinity chromatography).

**Figure 5:**
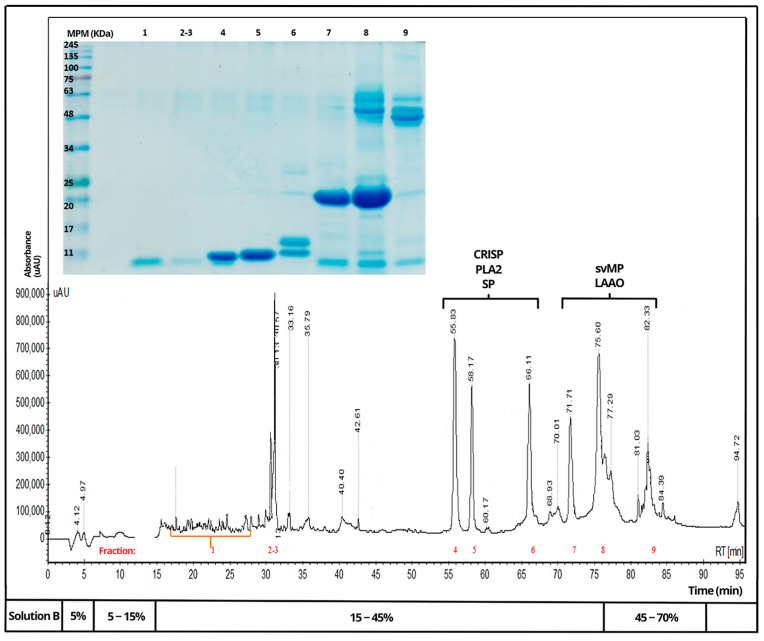
Reverse-phase HPLC separation of the venom proteins from *Bothrops asper*. Insert: SDS–PAGE of the isolated chromatographic fractions run under non-reducing conditions. (Red numbers represent the fractions selected for SDS—PAGE and affinity chromatography).

**Figure 6:**
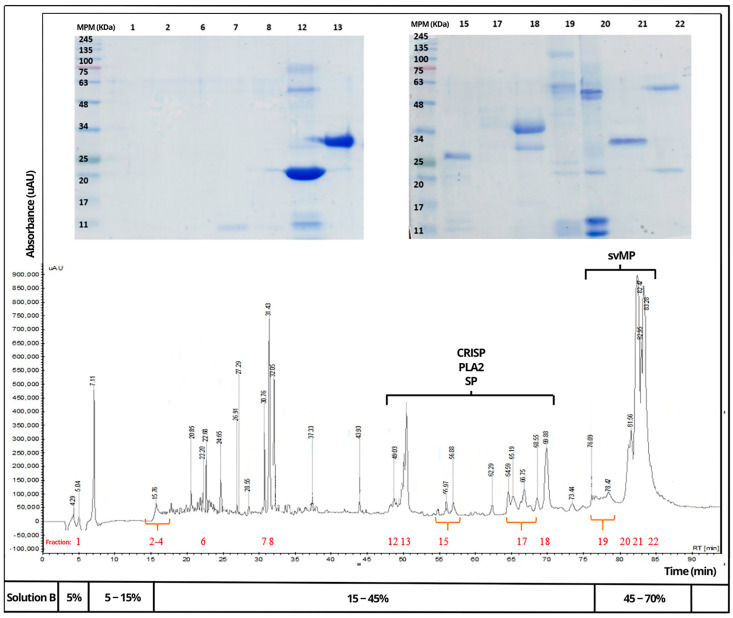
Reverse-phase HPLC separation of the venom proteins from *Bothrops atrox*. Insert: SDS–PAGE of the isolated chromatographic fractions run under non-reducing conditions. (Red numbers represent the fractions selected for SDS—PAGE and affinity chromatography).

Venoms of *B. rhombeatus* (Figure 4), *B. asper* (Figure 5), and *B. atrox* (Figure 6) were analyzed by reverse-phase HPLC fractionation and SDS-PAGE following the suggestions from researchers considering the protein characterization of the *Viperidae* family and specifically the *Bothrops* genus in Latin America [15,16], and because of the previous analysis of a large diversity of viperid venoms, rapid comparison of toxin profiles and classification of chromatographic fractions into protein families was possible without the need for extensive structural characterization [6].

Chromatograms of the three venoms showed that they share similar components within some protein families, mainly cysteine-rich secretory proteins (CRISPs), phospholipase A2 (PLA2), serine proteases (SPs), metalloproteinases (svMPs), and L-amino acid oxidases (LAAOs). In contrast, it was observed that the venom of *B. rhombeatus* presented a higher abundance of svMP III and I and PLA2, while a slight decrease in the concentration of svMP III and thrombin-like proteins was observed in the chromatograms of *B. atrox*, *B. asper*, and *B. atrox.*

### 2.2. Immunochemical Recognition

In ELISA tests, the level of recognition was expressed as a titer, defined as the concentration (μg mL) of antivenom required to achieve half of the maximum response (measured as Abs 405 nm) (Table 1).

ELISA assays showed that a higher concentration of INS antivenom was required to achieve half the maximum recognition response to *B. rhombeatus* venom compared to AVP-T antivenom. INS recognized *B. asper* and *B. atrox* venom, but it required higher concentrations to recognize the latter (Table 1).

The AVP-T antivenom required the lowest concentration to recognize the components of *B. atrox* venom, followed by *B. rhombeatus* venom, and finally *B. asper* (Table 1 and Figure 6). Statistical analysis with ANOVA showed statistically significant differences between the antivenoms concerning the amount of each antivenom required to reach half of the maximum recognition response to the antivenoms, performing the analysis by blocks according to each antivenom. With a calculated F value greater than the critical F and a *p* less than 0.05, the hypothesis of equality with 95% confidence was rejected.

The ANOVA results indicate that, despite the observed immunochemical recognition, in clinical practice, different amounts of both antivenoms are required to achieve 50% recognition of a specific venom, confirming the fact that one vial of antivenom does not recognize the same amount of venom for an entire genus, as currently detailed in the inserts of the drugs in question, and furthermore, the need to promote region-specific antivenoms is indisputable since there should be more direct recognition than cross-reactivity. Although greater recognition by the homologous antivenom is expected, it is possible that a venom to which it is not immunized has a higher proportion of some well-recognized proteins and is, therefore, better recognized than the homologous antivenom, which has been described in different studies with *Bothrops* venom [17,18].

**Figure 7:**
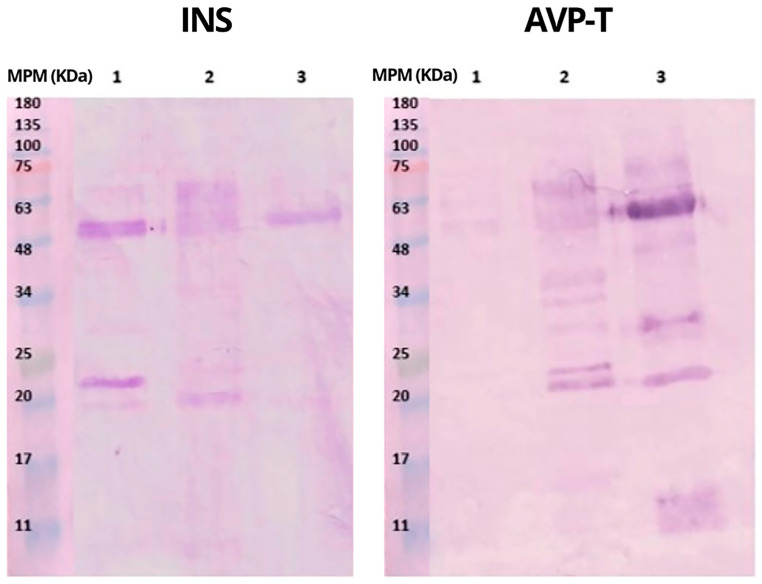
Western blot recognition of INS and AVP-T towards venoms. 1: *B. rhombeatus;* 2: *B. asper;* 3: *B. atrox;* MPM: molecular weight markers; INS: antivenom of National Institute of Health; AVP-T: antivenom Antivipmyn-Tri.

Western blot (Figure 7) evidenced immunochemical recognition of INS and AVP-T antivenoms; however, an adequate recognition of the protein components of *B. rhombeatus* by AVP-T was not shown. On the other hand, both antivenoms demonstrated good recognition of *B. asper* and *B. atrox* venoms.

These results show the possible recognition of the protein fractions of the three venoms through the location of the bands. With AVP-T antivenom, mild-intensity recognition bands were observed around 50 kDa, possibly svMP III of *B. rhombeatus* venom. From *B. asper* venom, bands between 20 and 75 kDa, possibly svMP I and III, were observed, while for *B. atrox* venom, recognition around 48–50 kDa was observed, together with other bands of lower intensity of 75, 35, 25, 20, and 11 kDa, possibly svMP III and I and PLA2, respectively.

The INS antivenom also showed protein recognition in all three venoms. For the venoms of *B. rhombeatus* and *B. asper*, recognition of bands at 48 and 25 kDa, possibly svMP III and I, was observed, respectively; meanwhile, for the venom of *B. atrox*, recognition of a band around 50 kDa, possibly svMP III, was observed.

Comparing the results of this experiment with those obtained in the ELISA assays, it can be inferred that INS requires more antivenom to have a 50% recognition level and binds mainly to the svMP I and III fractions of *B. rhombeatus* venom, while AVP-T requires less antivenom to obtain the same recognition.

Both antivenoms were similar in terms of recognition of svMP III and I of *B. asper*; however, in the ELISA results, a statistically significant difference was found in the amount of antivenom required for this recognition. It can be inferred that these antivenoms can easily identify *B. asper* proteins, possibly secondary to the similarity of these proteins between species of the same genus reported by some researchers [19,20,21]. The INS antivenom did not recognize the 11 kDa fractions, possibly the PLA2 of *B. atrox*. AVP-T recognized the highest amount of *B. asper* and *B. atrox* venom fractions, similar to findings by other authors [22,23]. 

Affinity chromatography evidenced that both antivenoms were effectively bound to fractions isolated from *B. rhombeatus* and *B. asper* venoms. INS bound to *B. rhombeatus* venom fractions at 94.2% while AVP-T bound at 90.4%. For *B. asper* venom as a control, INS had 92.7% coupling while AVP-T had 96.6% (Table 2), in concordance with Mora-Obando’s findings (Table 1) [19].

**Figure 8:**
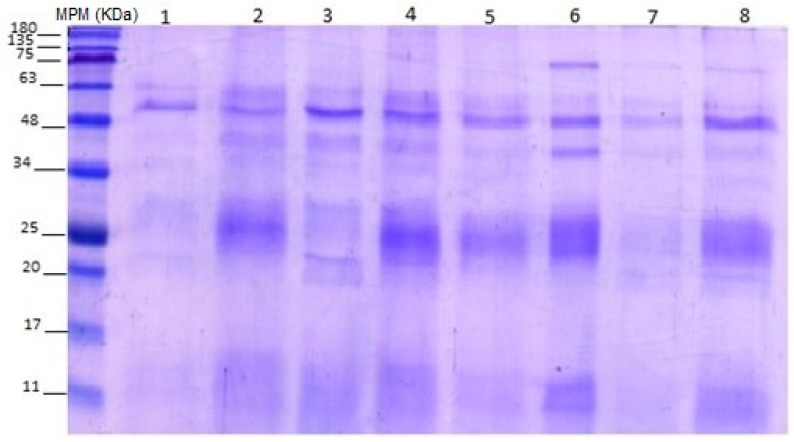
Electrophoresis of Ag-Ac coupled fraction. MPM: molecular weight markers. 1: INS + *B. rhombeatus* fractions 3–7; 2: INS + *B. rhombeatus* fractions 17–23; 3: AVP-T + *B. rhombeatus* fractions 3–7; 4: AVP-T + *B. rhombeatus* fractions 16–22; 5: INS + *B. asper* fractions 4–10; 6: INS + *B. asper* fractions 16–28; 7: AVP-T + *B asper* fractions 3–7; 8: AVP-T + *B. asper* fractions 19–27.

Considering the above tests, it can be deduced that the two antivenoms recognized most of the fractions of *B. rhombeatus* and *B. asper* venoms; however, both required high concentrations to achieve immunochemical recognition. The coupling of the antivenoms to the venoms was observed in the corresponding electrophoresis (Figure 8). Recognition of the protein fractions of the venom of *B. rhombeatus* and *B. asper* with both antivenoms was found, possibly through heterologous recognition since many fractions of the venoms are similar between the species.

In Figure 8, columns 1 to 4 correspond to fractions recognized in the *B. rhombeatus* venom. Columns 1–2 correspond to INS and columns 3–4 to AVP-T. Column 1 (INS + *B. rhombeatus* fractions 3–7) had the lowest recognition compared with columns 2 to 4. On the other hand, 5 to 8 correspond to fractions recognized in B. asper venom. Column 7 (AVP-T + *B asper* fractions 3–7) showed the lowest recognition compared with the others. Both antivenoms were coupled to svMP I and svMP II with defined bands, while for PLA2, a better coupling signal with AVP-T was observed. These results suggest a high affinity of the two antivenoms to svMP I and II of *B. rhombeatus*. For *B. asper* venom, better binding was observed for most of the protein fractions to both antivenoms. Subsequently, Table 3 shows an association of the fractions possibly recognized by the antivenoms according to the intensity level of the bands observed in electrophoresis. CRISP, svMP I, and SP proteins are found in a range between 20 and 25 kDa; therefore, differences found in the antivenom couplings for these proteins were measured together. 

The intensity of the band’s electrophoretic coloration was measured subjectively and Figure 8 demonstrated that the coupling of the collected second fraction by HPLC had a higher intensity than the first ones. CRISP, svMP I, and SP are located in the 20–25 kDa range, which suggests, because of the band’s intensity, a high coupling of both antivenoms with these proteins of *B. rhombeatus* and *B. asper* venoms (Table 3).

INS and AVP-T antivenom recognized the *B. rhombeatus* venom fractions but to different extents, requiring more INS antivenom to have 50% immunochemical recognition (ED50). Similarly, AVP-T recognized svMP III fractions from *B. rhombeatus* venom and bound 90% of the venom used; however, it required less antivenom and showed a higher number of bound fractions in electrophoresis following affinity chromatography.

It should be recalled that both antivenoms presented a possible cross-reactivity coupling, since neither was elaborated with *B. rhombeatus* venom, and recognized some fractions of the venom mentioned previously, which are not necessarily those that present the greatest clinical effect or severity in ophidiotoxicosis, for which reason, it is not possible to conclude with certainty which antivenom is best for counteracting the clinical effects of *B. rhombeatus* venom, and it is necessary to perform a complete toxicological characterization for this venom.

### 2.3. Median Lethal Dose of the Venoms 

With respect to the median lethal doses (LD50) of the venoms evaluated, the highest lethality was found in the venom of *B. asper* with 6.42–6.7 mg/kg. Table 4 specifies the LD50 for the venoms studied.

### 2.4. Median Effective Dose of the Antivenoms

Table 5 shows the results of the median effective dose (ED50) corresponding to the volume of antivenom necessary to neutralize 1 mg of theoretical venom. According to what is reported by each of the commercial companies, the AVP-T antivenom vial has the capacity to neutralize 30 mg of *Bothrops* venom, while each INS vial can neutralize the 70 mg vial [11,12]. In this study, the neutralizing capacity for *B. rhombeatus* venom was calculated to be 33 mg and 14 mg for each vial of INS and AVP-T antivenom, respectively (except for the IP inoculation route). It is important to consider that these calculations are based on IP LD50 and that the application of antivenoms as medical treatment must be performed intravenously, so this consideration must be taken into account before thinking about an extrapolation to clinical use.

Table 5 shows a comparison of the neutralizing capacity of each antivenom, whose measurements were adjusted according to the amount of antivenom protein. In addition, the amount of venom neutralized was described in three different measurement units. The results of the neutralization of *B. asper* and *B. atrox* venom showed that INS required a lower concentration of antivenom compared to AVP-T. It was also found that AVP-T obtained the best immunochemical recognition titers for the fractions of the three venoms studied.

## 3. Discussion

In general, the protein profiles analyzed for the venoms of the three species are similar, with proteins of high, medium, and low molecular weight. The electrophoretic profile of *B. rhombeatus* was very similar to that found for the venom of *B. asper* [20,24,25] where 13 to 15 kDa may correspond to PLA2 observed in all electrophoresis experiments; the fractions of 20 to 25 may correspond to svMP I, and those around 45 to 48 kDa to svMP III, according to what has been found by several authors for *B. asper.*

It was found that each 10 mL vial of INS antivenom has a neutralizing capacity of 66.6 mgV/10 mL for *B. asper*, 55.5 mgV/10 mL for *B. atrox*, and 13.3 mgV/10 mL for *B. rhombeatus*. This result does not correspond to what is described in the product’s summary for the public, because the neutralizing capacity for *Bothops sp.* is reported to be 70 mg, but the same amount of poison is not neutralized for all species of that genus. Likewise, it was found that each 10 mL vial of AVP-T antivenom has a neutralizing capacity of 27.7 mgV/10 for *B. asper* mL, 21.2 mgV/10 mL for *B. atrox*, and 14.7 mgV/10 mL for *B. rhombeatus*. In the same way as for INS, the product’s summary for the public describes a neutralizing capacity for the *Bothops* sp. Of 30 mg, without discriminating by species, generating inaccuracies with the other species. Therefore, both antivenoms present similarities between the neutralizing capacity calculated in our trial and reported in the products’ summaries for the public for the *B. asper* species; however, it does not apply to the other species tested in this trial.

Protein similarities and abundances between *B. asper* venoms from different regions and other species were found in the literature [18,19,25,26]. This relationship may be useful to explain why AVP-T required a lower concentration to recognize the components of *B. atrox* venom, followed by *B. rhombeatus* venom and finally *B. asper* venom. It should be remembered that in this experiment, complete venom was used and that none of the antivenoms was elaborated on with *B. rhombeatus* venom, so it is inferred that there is cross-recognition between some similar fractions of the venoms.

Better recognition of AVP-T was found towards *B. atrox* venom fractions and *B. rhombeatus* venom fractions, which correlates with what was observed in neutralizing capacity assays where both antivenoms neutralized the challenged venom dose. In general, AVP-T showed better recognition for most of the fractions of *B. asper* and *B. atrox* venoms compared to INS. These results are similar to those obtained by other researchers [22,23].

Both antivenoms were similar in terms of recognition of the svMP III and I of *B. asper*; however, ELISA results showed a statistically significant difference in the amount of antivenom required, inferring that these antivenoms were made in part with *B. asper* venom. It has been observed in other studies that this is a species that presents similarities in the composition of its venom, despite the species-specific variability and that observed in some regions [21].

We reiterate the importance of corroborating the results via various essays because only the neutralizing capacity is not indicative of antivenom’s effectiveness and specificity. It is necessary to check the recognition of antivenoms’ protein fractions by ELISA and Western blot. We suggest that every antivenom producer perform neutralizing capacity and specificity trials by ELISA or Western blot. 

Taking into account that recognition does not imply neutralization of the fractions with greater activity in the pathophysiology of ophidiotoxicosis, the results of the ELISA test, together with those of ED50, showed that to neutralize the lethal dose of *B. rhombeatus* venom, a higher concentration of both INS and AVP-T antivenom was required. This result has clinical implications, where using a large amount or concentration of heterologous proteins for neutralization generates a great risk for patients. Obando et al. (2021) compared the neutralizing capacity of several antivenoms for the venoms of *B. asper*, *B. rhombeatus,* and *Bothrops yerbei*. They found that the ED50 of INS antivenom for *B. asper*’s venom was 5 mgV/mlAV, which is similar to our trial result of 6.6 mgV/1 mL (Table 5). The results of INS antivenom for *B. rhombeatus*’s venom was 5.6 mgV/mlAV (5.0–6.3), which is not consistent with our result of 1.3 mgV/1 mL (Table 5). These differences between the results may be due to several factors including biological factors of the reference animals, but they also highlight the need to specify the neutralizing capacity by species and not generalize by genus.

The results obtained in the immunochemical affinity chromatography can be correlated with those obtained by some authors, such as Bourke and collaborators, who evaluated the immunochemical recognition of snake venoms with several antivenoms, including AVP-T, and obtained adequate results of neutralization and affinity for *B. asper* [22].

Western blotting results allowed the electrophoretic identification of the fractions recognized by each antivenom, finding less visualization of bands of *B. rhombeatus* venom with AVP-T compared to INS; however, when confronted with *B. asper* and *B. atrox*, the low specificity of both antivenoms towards *B. rhombeatus* is evidenced. Furthermore, with the remarkable affinity of AVP-T to the venoms of *B*. *asper* and *B. atrox*, it is inferred that these two venoms were used for its elaboration.

According to immunorecognition analysis by RP-HPLC and affinity immunochromatography, it was shown that both antivenoms had a high affinity (more than 90%) to the complete venom of *B. rhombeatus* despite their low specificity, which is explained by the high concentration of antivenom that was required for the recognition of structural epitopes and its effect on cross-reactivity for neutralization. This technique and analysis have been demonstrated in recent trials to prove specificity in antivenom recognition, where the percentage of immunorecognition was obtained by the integration of RP-HPLC profiles of retained and non-retained fractions [19]. 

An intraperitoneal (IP) LD50 of *B. rhombeatus* has been reported at 54.9 μg for mice (36–83.8), equivalent to 3.05 mg/kg [19], which is lower than that found in our study at 6.6 mg/kg, possibly secondary to species intraspecific variability. Our result was found to be similar to that reported for *B. rhombeatus* [27].

In multiple studies evaluating the neutralizing capacity of antivenoms at the preclinical level, it has been found that polyvalent antivenoms are capable of recognizing some fractions of snake venoms from other regions, including venoms of species other than those used in their production, and may also have adequate neutralizing capacity, although they require more antivenom to do so [28,29,30]. This allows us to infer that there are some protein fractions that are similar among the venoms, which are not necessarily the most abundant, immunogenic, or those that cause the greatest clinical effect. For this reason, it is necessary to increase the quality of the antivenoms to the extent possible, making them more specific and related to the fractions of interest, which means a smaller quantity of antivenom can be used to recognize the epitopes and neutralize them quickly.

## 4. Conclusions

INS and AVP-T antivenoms can neutralize the lethal effect of three LD50 of *B. rhombeatus* venom via IP, but with different amounts of antivenom, which correlates with the percentage of affinity coupling, concluding that some of the fractions with pathophysiological lethal action were recognized by cross-reactivity and neutralized. It should be taken into account that this amount of antivenom required to neutralize three LD50 was developed via IP, and there may be differences when it is developed via IV, with a lower amount of antivenom required via IV than via IP. When compared with the results obtained in this research, they are congruent with the neutralizing capacity described by the inserts of the two antivenoms (despite the consideration of the inoculation route), since they infer that they neutralize the same amount of venom at a general level for the whole genus *Bothrops.*

To evaluate the efficacy of antivenoms, the neutralizing capacity or ED50 is used, but what has been demonstrated in this study is that the greatest importance lies in the antivenoms being specific and related to the fractions of clinical interest, taking into account the immunogenicity, their abundance in a particular venom, and the toxic effect of the fractions. The demonstrated specificity of antivenoms to *B. rhombeatus* venom translates into a greater number of vials to be used to look for a cross-reactivity effect and achieve neutralization.

## 5. Materials and Methods

### 5.1. Venoms

The venom of *B. rhombeatus* was obtained from the only place where it was under professional care at the time the study was initiated, the Fundación Zoológica de Cali (FZC). One adult was found in the Municipality of Cali, Valle del Cauca, Colombia, by the Fundación Zoológica de Cali (FZC). Venom was manually extracted three times, freeze-dried, and kept at −80 °C. Equal parts of the collected samples were mixed, distributed in 5 cryovials, and assigned lot number FZC 041119. Other venoms were included to obtain comparison in the assays, which were supplied by the Institute of Biotechnology (IBT) of UNAM: *B. asper* lot (10 November 2017) and *B. atrox* lot 30 × 8901. All vials and venom aliquots were kept at −20 °C and, at the time of testing, were kept refrigerated.

### 5.2. Antivenoms

Vials of polyvalent antiophidic antivenom were purchased from INS Lot 18SAP02, expiration date October 2021, INVIMA 2012M-0013350. Vials of AVP-T polyvalent antiophidic faboterapic antivenom were purchased, Lot B-7B-32, expiration date April 2021. AVP-T was reconstituted in 5 mL of PBS. INS did not require saline because it is liquid. the 280 nm Absorbance method for INS and AVP-T antivenoms was used to calculate the protein concentration, and a correction factor 1.4 of IgG weight was used. Dilution for INS was 1:50 and the average protein concentration was 44.3 mg/mL, while AVP-T dilution was 1:100 and presented an average protein concentration of 12.6 mg/mL.

Protein quantification of antivenoms was performed at the beginning of the investigation and before carrying out each experiment. Initial measurements were made by the 280 nm Absorbance method. The protein concentration results are shown in Table 6. For a detailed review of the methodology, see Supplementary Materials.

Information about the neutralizing capacity is described in Table 7 and comes from of label antivenom as follows:

### 5.3. Electrophoresis (SDS-PAGE)

Gels were prepared in duplicate under non-reducing and reducing conditions with β-mercaptoethanol for all venoms at a concentration of 2 μg/μL; 15 μg and 30 μg of venom were loaded per lane. For the post-RP-HPLC fractions, 4–20% Tris-Glycine MPM was used. They were dried and resuspended in PBS 1X depending on the collected volume of the fraction. A 10 μg amount of venom was loaded in each lane. For the antivenoms, MPM Tris-Glycine 4–20% was used. For INS antivenom, a 1:50 dilution was performed, and for AVP-T, a 1:100 dilution was performed loading a maximum of 15 μg per lane. The technique was developed under non-reducing and reducing conditions with β-mercaptoethanol.

### 5.4. RP-HPLC Reverse-Phase High-Performance Liquid Chromatography

RP-HPLC was performed using the Galaxie Chromatography Data System software with a C18 column (250 mm × 4.6 mm). XCrhroma^TM^ was used for the venoms *B. rhombeatus, B. asper, and B. atrox*. Between 2 and 3 mg of each venom was used in 300 μL in 1.2 mL of water with 0.1% TFA and centrifuged at 14,000 rpm for 3 min. A gradient of 5% solution B was used in the first 5 min, 5–15% B from 5 to 15 min, 15–45% B from 15 to 75 min, and 45–70% from 75 to 90 min, for a total run of 90 min at a flow rate of 1 mL min.

### 5.5. Titers by ELISA

Plates were sensitized with 100 μL of complete venom in each well at a concentration of 5 μg mL; 200 mL of ELISA blocking solution was used for 2 h at 37 °C. A total of 150 μL of antivenom was used in the first column of wells at a concentration of 700 μg mL, and serial 1:3 dilutions were performed. Anti-horse IgG (H + L) for INS and anti-horse F(ab)2 for AVP-T, coupled to alkaline phosphatase at a dilution of 1:4000 KPL brand, were used as secondary antibodies. Samples were read at 10, 15, 20, and 30 min after incubation.

### 5.6. Western Blot

Fifteen percent SDS-PAGE and transfer analysis were performed under reducing conditions with β-mercaptoethanol. Each lane was loaded with 4 μg of each venom. Each antivenom was brought to a concentration of 200 μg/mL in 10 mL. Proteins from unstained gels were transferred to a nitrocellulose membrane treated with a goat anti-horse IgG (H + L) antibody at a 1:2000 dilution (KPL lot N°120607)-.

### 5.7. Affinity Chromatography

Affinity chromatography is based on a specific and reversible interaction between a protein and a molecule coupled to a porous matrix generally made of resin. The protein is considered a fining agent that will bind to a ligand found in the resin matrix. Affinity chromatography was performed to quantify the interaction between the specific antibodies present in the INS and AVP-T antivenoms, against the proteins of the *B. rhombeatus* and *B. asper* venoms, according to the technique standardized by the IBT. Antivenom columns were created using 0.7 g of 4B-CNBr sepharose. In total, 1 mL of INS antivenom and 3 mL of AVP-T antivenom were dialyzed with 0.1 M NaHCO3. The resin was attached with 1.5 mg of *B. rhombeatus* venom for each of the columns at a concentration of 2 mg/mL, and *B. asper* venom at the same concentration was used as a control. The visualization of the experiment’s affinity was performed by polyacrylamide gel electrophoresis of venom fractions coupled to antivenoms. 

### 5.8. In Vivo Biological Activities

The assays were performed within the framework of the projects approved by the Bioethics Committee of the Faculty of Veterinary Medicine and Zootechnics (MVZ) of the UN [CB-FMVZ-UN-019-19]. Albino mice, Mus musculus, strain CD1, weighing between 18 and 20 g, males and females, from and maintained at the Biotherium of the Institute of Biotechnology of the UNAM were used.

#### 5.8.1. Median Lethal Dose

The LD50 was calculated as mg/kg according to the calculated protein for each antivenom and mouse weight (approximately 20 g). Groups of 3 mice were inoculated with different amounts of venom. Each inoculation mixture contained venom and saline to complete a volume of 0.5 mL per animal. Intraperitoneal (IP) inoculation was performed. For each species of snake in this trial, six groups of venom doses were previously established: 50 μg, 70 μg, 90 μg, 110 μg, 130 μg, and 150 μg. Subsequently, doses were adjusted to test each venom according to the aforementioned venom doses, where a lethality curve of 0–100% was obtained. Mortality was observed and noted at 24 h post-inoculation.

#### 5.8.2. Median Effective Dose

Different doses of antivenom were inoculated to groups of 3 mice, with 3 LD50 of venom and saline solution to complete a volume of 0.5 mL per animal; the mixture was incubated for 30 min at 37 °C. Inoculation was performed via IP. This test was performed for the two antivenoms with each of the venoms. Survival was observed and recorded at 24 h post-inoculation. The amount of antivenom necessary to neutralize 1 mg of venom (mlAV/mgV) was calculated according to the amount of venom on three (3) DL50. The amount, in mg, of venom neutralized by 10 mL of antivenom was calculated according to the antivenom necessary to neutralize 1 mg of venom (mlAV/mgV).

### 5.9. Statistical Analysis

ELISA immunochemical recognition data were analyzed by one-way analysis of variance (ANOVA) using an F-test with 95% confidence; differences with *p* values less than 0.05 were considered statistically significant. A non-linear sigmoid regression-type dose–response dependent variable was used to calculate the LD50 and ED50 with GraphPad Prism software version 6.0b.

## 6. Limitations

In our trial, the limitation was that venom could only be obtained from one (1) specimen of the *B. rhombeatus* species. In Colombia, according to decree 1376 of 2013, “the collection of wild specimens of biological diversity for non-commercial research purposes” must be carried out through a national institutional agreement; therefore, to make research more affordable, agreements are made with zoos that have permission and wild species in captivity. For this reason, an agreement was reached with the only zoo in Colombia that had specimens of *B. rhombeatus* at the date of research.

## Figures and Tables

**Table 1:** ELISA recognition of INS and AVP-T towards venoms. ELISA recognition level, expressed as the concentration of antivenom (μg/mL) required to achieve half the maximum response (Abs 405 nm).

Venom Species	Antivenom	μg/mL	95% Confidence Interval
*B. rhombeatus*	INS ^1^	4.089	3.633–4.602
	AVP-T ^2^	2.513	2.097–3.011
*B. asper*	INS	1.982	1.830–2.147
	AVP-T	3.993	2.743–5.812
*B. atrox*	INS	5.183	4.228–6.352
	AVP-T	1.647	1.441–1.883

^1^ INS: antivenom of National Institute of Health; ^2^ AVP-T: Antivipmyn-Tri antivenom.

**Table 2:** Quantification of INS and AVP-T coupling by affinity chromatography of the isolated fractions from *B. rhombeatus* and *B. asper* venoms.

Venom Species	Antivenom	Non-Joined Venom (mg)	Joined Venom (mg)	Coupling Percentage (%)
*B. rhombeatus*	INS	2.2	37.7	94.2
	AVP-T	3.5	32.5	90.4
*B. asper*	INS	2.9	37.1	92.7
	AVP-T	1.2	34.8	96.6

**Table 3:** Association of the potential immunochemical recognition of antivenoms.

Electrophoresis Bands (kDa)	Protein Fractions Possibly Recognized	AVP-T’s Level of Recognition (Intensity)	INS’s Level of Recognition (Intensity)
10–15	PLA_2_	Middle	Middle
20–25	CRISP, svMP I, and SP	High	High
48–55	svMP II	High	High
60–90	svMP III	High	--

**Table 4:** Comparison of LD_50_ of venoms.

Venom	Inoculation Pathway	LD50 μg/Mouse CD1 ^1^	LD50 mg/kg ^2^	95% CI ^3^
*B. rhombeatus*	IP ^4^	132.7	6.6	119.9–146.9
*B. asper*	IP	128.1	6.4	120.9–135.7
*B. atrox*	IP	134.7	6.7	121.9–148.8

^1^ LD_50_ μg/mouse: micrograms of venom needed to kill 50% of experimental mice (CD1, 20 g); ^2^ LD_50_ mg/kg: milligrams of lethal venom per kilogram of body weight; ^3^ 95% CI: 95% confidence interval; ^4^ IP: intraperitoneal.

**Table 5:** Effective dose of INS and AVP-T for the different venoms.

Venom Species	Antivenom	μlAV/3LD_50_^1^	95% CI ^2^	mlAV/mgV ^3^	mgV/10 mL ^4^
*B. rhombeatus*	INS	119.7	116.3–123.2	0.30	33.3
	AVP-T	270.2	258.2–282.7	0.68	14.7
*B. asper*	INS	62.7	56.04–70.25	0.15	66.6
	AVP-T	143.8	112.9–168.4	0.36	27.7
*B. atrox*	INS	75.15	72.3–78.06	0.18	55.5
	AVP-T	190.2	164.1–220.4	0.47	21.2

^1^ μlAV/3LD_50_: microliters of antivenom necessary to neutralize 3 lethal average doses; ^2^ confidence interval; ^3^ mlAV/mgV: milliliters of antivenom needed to neutralize 1 mg of venom; ^4^ mgV/10 mL: milligrams of venom neutralized by 10 mL of antivenom IP.

**Table 6:** Protein quantification of antivenoms by 280 nm Absorbance method.

Antivenom	280 nm Absorbance
INS	44.3 mg/mL
AVP-T	12.6 mg/mL

**Table 7:** Neutralizing capacity according to the summary for the public.

Antivenom	Neutralizing Capacity	Liquid Amount of Antivenom
INS	10 mg of *Crotalus* sp. venom and 70 mg of *Bothrops* sp. venom. By cross-reaction, each vial neutralizes at least 15 mg of *Lachesis muta* venom (Amazonian ecoregion) and 50 mg of *Lachesis acrochorda* venom (Pacific and Inter-Andean valley ecoregion) ^1^	10 mL
AVP-T	30 mg of *Bothrops* sp. venom, 15 mg of *Crotalus* sp. venom, and 7 mg of *Lachesis* sp. venom ^2^	10 mL

^1^ Instituto nacional de Salud (INS). Polyvalent antivenom serum VI:06 summary for the public. 2017. Available from www.ins.gov.co (accessed on 21 November 2019); ^2^ Bioclon. Polyvalent antivenom fabotherapic antivipmyn. 2017. Available from: https://www.invima.gov.co/atencion-al-ciudadano/consulta-avanzada-registros-sanitarios (accessed on 3 December 2019).

## Data Availability

Data are contained within the article.

## Supplementary Materials

The following supporting information can be downloaded at https://www.mdpi.com/article/10.3390/toxins16030152/s1. Figure S1: BAC standardization curve for antivenom quantification; Figure S2: Bradford standardization curve for antivenom quantification. AVP-T antivenom (a) and INS antivenom (b); Figure S3: Bradford standardization for antivenom quantification. AVP-T antivenom standardization by Bradford (a) and INS antivenom standardization by Bradford (b); Table S1: BSA standard dilution preparation (2 mg/mL), for Bradford.
