# Supplementary material for: Immunochemical Recognition of Bothrops rhombeatus Venom by Two Polyvalent Antivenoms
Source: Toxins (Basel). 2024 Mar 15;16(3):152. doi: 10.3390/toxins16030152 (PMC10975100; doi:10.3390/toxins16030152)
Supplement: Supplementary file 1 [file toxins-16-00152-s001.zip › toxins-2877567-supplementary.pdf]

Supplementary Materials: Immunochemical Recognition of *Bothrops rhombeatus* Venom by Two Polyvalent Antivenoms

1. Quantification by 280nm Absorbance

An antivenoms protein quantification were performed at the beginning of the research and after each experiment thought 280 nm absorbance method. AVP-T was reconstructed in 5ml of PBS and 1:100 dilution was performed. INS had a presentation of 10ml vial; therefore, it was not reconstructed; and 1:50 dilution was performed. It was adjusted according to immunoglobulin correction factor (1.4), where results of 12,6 mg/ml and 44,3 mg/ml were obtained for AVP-T and INS, respectively.

2. Quantification by Bicinchoninic acid (BAC)

A calibration curve was generated as a reference for antivenom protein quantification. INS antivenom was used at a 1:200 dilution while AVP at a 1:50 dilution. The imagine shows the trend line along with the equation of the line and the respective correlation coefficient, which is close to 1.

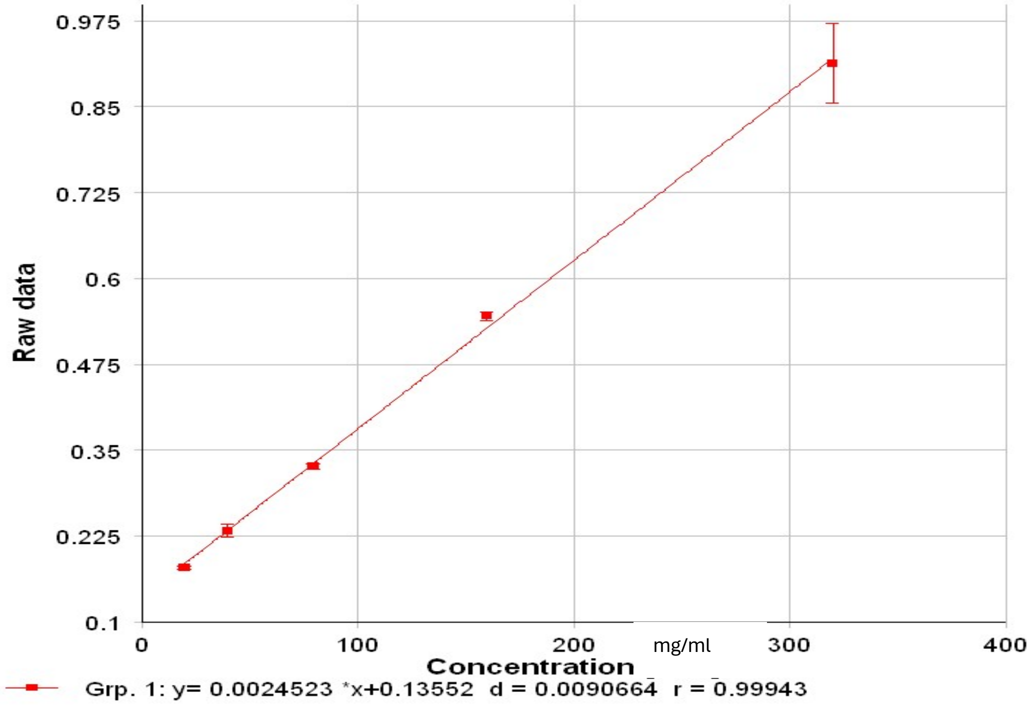

Figure S1. BAC's standardization curve for antivenoms quantification.

3. Quantification by Bradford

A calibration curve was obtained for this method. The curve was generated using 4 concentrations of BSA, the reading was measured at a wavelength of 595nm. For the INS antivenom, a serial dilution of 1:100 – 1:200 – 1:400 – 1:800 was carried out; while for the antivenom antivipmyn a serial dilution of 1:15 – 1:20 – 1:25 – 1:30 was carried out.

Figure 2 shows the trend line, the equation of the straight line and the respective correlation coefficient, which is close to 1. For the calculations of the antivenoms, the molar extinction coefficient of immunoglobulins G ($\varepsilon = 1.4$).

Table S1. BSA's standard dilution preparation (2 mg/ml), for Bradford.

Antivenoms	Protein concentration	Dilution	BSA μl	Saline solution μl
Instituto nacional de salud (INS)	5.4 mg/ml	1:15	33.5	466.5
		1:20	25	475
		1:25	20	480
		1:30	16.5	483.8
Antivipmyn (AVP-T)	40.2 mg/ml	1:100	5	495
		1:200	2.5	497.5
		1:400	1.25	498.75
		1:800	0.625	499.37

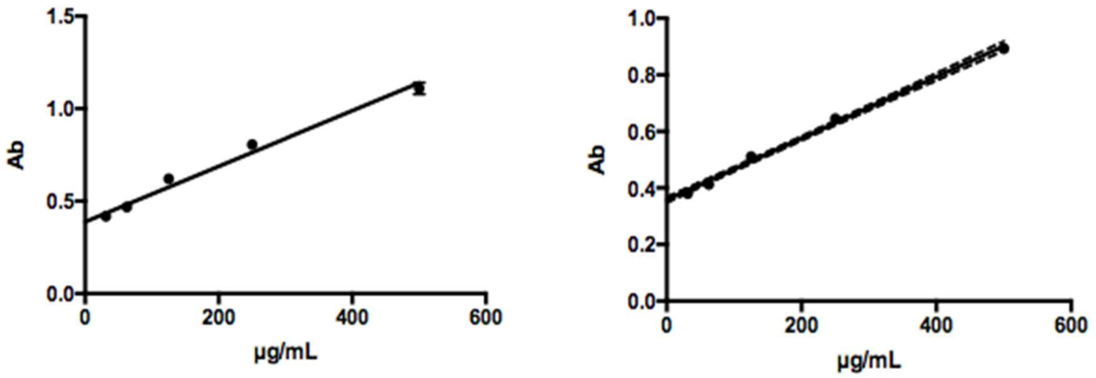

(**a**) (**b**)

Figure S2. Bradford's standardization curve for antivenoms quantification. AVP-T antivenom (**a**) and INS antivenom (**b**).

Linear reg.<br>Tabular results		A<br>Ab<br>Y
1	Best-fit values	
2	Slope	0.001502 ± 5.259e-005
3	Y-intercept when X=0.0	0.3880 ± 0.01239
4	X-intercept when Y=0.0	-258.3
5	1/slope	665.6
6	95% Confidence Intervals	
7	Slope	0.001391 to 0.001614
8	Y-intercept when X=0.0	0.3618 to 0.4143
9	X-intercept when Y=0.0	-294.4 to -226.8
10	Goodness of Fit	
11	R square	0.9808
12	Sy.x	0.03828
13	Is slope significantly non-zero?	
14	F	816.0
15	DFn, DFd	1.000, 16.00
16	P value	< 0.0001
17	Deviation from zero?	Significant
18	Data	
19	Number of X values	6
20	Maximum number of Y replicates	3
21	Total number of values	18
22	Number of missing values	0
23		
24	Equation	Y = 0.001502*X + 0.3880

(**a**)

Linear reg.<br>Tabular results		A<br>Ab<br>Y
1	Best-fit values	
2	Slope	0.001088 ± 2.084e-005
3	Y-intercept when X=0.0	0.3580 ± 0.004910
4	X-intercept when Y=0.0	-329.1
5	1/slope	919.3
6	95% Confidence Intervals	
7	Slope	0.001044 to 0.001132
8	Y-intercept when X=0.0	0.3476 to 0.3684
9	X-intercept when Y=0.0	-351.1 to -308.8
10	Goodness of Fit	
11	R square	0.9942
12	Sy.x	0.01517
13	Is slope significantly non-zero?	
14	F	2724
15	DFn, DFd	1.000, 16.00
16	P value	< 0.0001
17	Deviation from zero?	Significant
18	Data	
19	Number of X values	6
20	Maximum number of Y replicates	3
21	Total number of values	18
22	Number of missing values	0
23		
24	Equation	Y = 0.001088*X + 0.3580

(**b**)

Figure S3. Bradford's standardization for antivenoms quantification. AVP-T antivenom standardization by Bradford (**a**) and INS antivenom standardization by Bradford (**b**).
